# Gender and Age-Specific Responses to Non-Invasive Body-Contouring Interventions and Their Impact on Body Composition—Pilot Study

**DOI:** 10.3390/nu17162639

**Published:** 2025-08-14

**Authors:** Raluca Maior, Florina Ruta, Mihail-Alexandru Badea, Calin Avram, Vladimir Bacârea

**Affiliations:** 1 Department of Research Methodology, George Emil Palade University of Medicine, Pharmacy, Science and Technology of Targu Mures, Gheorghe Marinescu Street No. 38, 540136 Targu Mures, Romania; raluca.maior@umfst.ro (R.M.); vladimir.bacarea@umfst.ro (V.B.); 2 Department of Community Nutrition and Food Safety, George Emil Palade University of Medicine, Pharmacy, Science and Technology of Targu Mures, Gheorghe Marinescu Street No. 38, 540136 Targu Mures, Romania; 3 Natural Skin, 540142 Targu Mures, Romania; 4 Department of Medical Informatics and Biostatistics, George Emil Palade University of Medicine, Pharmacy, Science and Technology of Targu Mures, Gheorghe Marinescu Street No. 38, 540136 Targu Mures, Romania; calin.avram@umfst.ro

**Keywords:** body composition, electrical muscle stimulation, ultrasound cavitation, non-invasive intervention

## Abstract

Background: Eximia is a non-invasive body-contouring technology combining ultrasound cavitation, radiofrequency, and vacuum suction to reduce adiposity. EMS Pro Bodytech delivers biphasic electrical impulses to stimulate muscular contractions and improve muscle performance. Methods: A 6-week observational study included 77 participants (58 women aged 28–55 and 19 men aged 20–49), who received twice-weekly sessions combining Eximia and EMS training. Anthropometric and body composition measurements were recorded before and after the intervention. Results: Participants showed reductions in fat mass (mean from 19.21 kg to 18.19 kg; SD from 8.23 to 8.42), BMI (mean from 26.03 to 25.68; SD from 4.26 to 4.16), and visceral fat index (mean from 4.97 to 4.74; SD from 2.88 to 2.99), alongside an increase in skeletal muscle percentage (mean from 37.34% to 38.3%, SD from 5.09 to 5.94). Statistical analysis revealed no significant differences in treatment response between genders (e.g., BMI: *p* = 0.080; fat-free mass: *p* = 0.089) or age groups (all *p* > 0.6), suggesting that the intervention was effective across demographics. Conclusions: The combined approach of Eximia body remodeling and EMS muscle stimulation led to measurable improvements in body composition, independent of age or gender. These findings support its potential as a non-invasive, inclusive strategy for body reshaping alongside peri-procedural dietary standardization.

## 1. Introduction

Maintaining an optimal body composition and an aesthetically pleasing appearance has become a central concern among adults, particularly after the age of 30, when a healthy lifestyle gains a dual significance—both functional and aesthetic. The adoption of balanced dietary habits, adequate sleep, proper hydration, and regular physical activity has been consistently associated with significant long-term health benefits [1,2].

Nevertheless, many individuals continue to experience localized adipose tissue deposits and persistent cellulite, particularly in areas such as the abdomen and thighs, despite adherence to health-promoting behaviors.

Against this backdrop, non-invasive body contouring therapies have gained increasing popularity due to their ability to reduce subcutaneous fat, improve dermal tone, and achieve visible aesthetic outcomes without the need for surgical intervention. Technologies such as the Eximia HR77 Platinum system integrate ultrasound cavitation, multipolar radiofrequency, and negative pressure vacuum, allowing for simultaneous action on adipose tissue and dermal structures [3,4,5]. In parallel, electrical muscle stimulation (EMS) has been recognized for its ability to induce deep, involuntary muscle contractions, recruiting up to 90% of muscle fibers, including those that are typically difficult to activate through voluntary exercise [6]. Evidence suggests that EMS can enhance muscle mass and strength, particularly when applied in short-term protocols [7].

Preliminary data indicate that combined interventions—integrating body contouring techniques with neuromuscular stimulation—may exert synergistic effects on body composition, potentially providing superior benefits compared to the isolated application of each modality [8]. However, the impact of such combined therapies on anthropometric parameters, as well as the influence of individual factors such as age and sex, remains insufficiently investigated in the current literature.

The aim of the present study is to evaluate the effectiveness of a combined protocol involving Eximia treatments (ultrasound and radiofrequency) and EMS on body composition parameters in adult subjects, while also examining potential variations in therapeutic response according to age and gender.

In light of the study’s objective to evaluate the impact of non-invasive interventions on body composition, a peri-procedural nutritional standardization protocol emphasizing controlled protein intake was implemented to mitigate the potential confounding effects of inter-individual dietary variability. By controlling pre- and post-session intake across all participants, the study ensured more accurate attribution of observed changes in muscle mass, hydration status, and basal metabolic rate to the intervention itself.

## 2. Materials and Methods

### 2.1. Study Design and Participants

This six-week observational cohort study pilot evaluated the combined effects of non-invasive body contouring and electrical muscle stimulation (EMS) on body composition. A total of 77 sedentary adults (aged 25–55 years) seeking weight and fat loss were recruited from the “Nutricare Anti-Aging” clinic in Târgu-Mureș, Romania.

Inclusion criteria required participants to report less than 2 h of structured physical activity per week and to maintain a predominantly sedentary occupational and leisure lifestyle. All participants provided written informed consent, and the study protocol received approval from the clinic’s ethics committee. As this was an exploratory pilot study, no formal sample size calculation was performed; the sample size was based on clinical feasibility and participant availability within the defined study period.

### 2.2. Intervention Protocol

**Figure 1:**
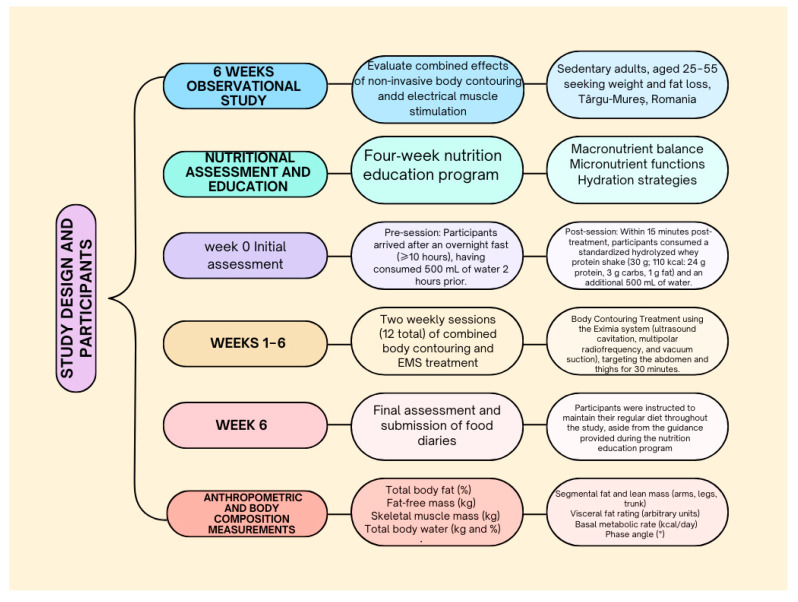
Intervention protocol stages.

The intervention consisted of the following protocol, implemented over six consecutive weeks: Week 0: Initial assessment; Weeks 1–6: Two weekly sessions (12 total) of combined body contouring and EMS treatment; Week 6: Final assessment and submission of food diaries (Figure 1).

### 2.3. Each Intervention Session Included

Body Contouring Treatment using the Eximia system (Milan, Italy) (ultrasound cavitation, multipolar radiofrequency, and vacuum suction), targeting the abdomen and thighs for 30 min.

EMS Training using the EMS Pro Bodytech device (Guangzhou, China) (20–100 Hz frequency; 300 μs pulse width) applied to abdominal and thigh muscles for 20 min at individually titrated intensities.

### 2.4. Peri-Procedural Standardization

Pre-session: Participants arrived after an overnight fast (≥10 h), having consumed 500 mL of water 2 h prior.

Post-session: Within 15 min post-treatment, participants consumed a standardized hydrolyzed whey protein shake (30 g; 110 kcal: 24 g protein, 3 g carbs, 1 g fat) and an additional 500 mL of water.

### 2.5. Nutritional Assessment and Education

Prior to the intervention, all participants completed a four-week nutrition education program consisting of four one-hour sessions focused on: macronutrient balance; micronutrient functions; hydration strategies.

To assess habitual dietary intake, participants were instructed to keep a three-day food diary, (two weekdays and one weekend day) at baseline (week 0) and again at week 6. These were reviewed in person by a registered dietitian and analyzed using Eatntrack software vers. 1.10.8 (Cluj-Napoca, Romania) to estimate: total daily energy intake (kcal); macronutrient distribution (% of total kcal); water consumption (mL).

### 2.6. Dietary Controls

Participants were instructed to maintain their regular diet throughout the study, aside from the guidance provided during the nutrition education program. Use of weight-loss supplements or major dietary changes led to exclusion from per-protocol analyses.

### 2.7. Physical Activity Assessment

To confirm sedentary status, participants completed the International Physical Activity Questionnaire–Short Form (IPAQ-SF) at baseline. The IPAQ-SF assesses weekly MET-minutes across walking, moderate, and vigorous activities. Participants reporting <600 MET-minutes/week—equivalent to <150 min of moderate–intensity physical activity—were classified as sedentary and eligible for inclusion.

Participants were instructed to abstain from vigorous physical activity for at least 48 h prior to each assessment.

### 2.8. Anthropometric and Body Composition Measurements

All measurements were performed at baseline and at week 6 under standardized conditions to minimize variability: Fasting: Overnight fast (≥10 h); Fluid intake: 500 mL of water 2 h prior to measurement; Caffeine and alcohol: Avoided for 12 h before testing; Bladder voiding: Within 30 min prior; Environment: Temperature-controlled room (22 ± 1 °C), assessments conducted between 07:00 and 10:00 a.m.

### 2.9. Anthropometric Measures

Height: Measured to the nearest 0.1 cm (Seca 217 stadiometer (Hamburg, Germany)).

Circumferences: Waist, hip, mid-upper arm, and mid-thigh circumferences measured in duplicate (Lufkin W606PM tape (Missouri City, TX, USA)); mean values used.

### 2.10. Body Composition Analysis

Using a multifrequency bioelectrical impedance analyzer (Tanita MC-780MA-N; Tokyo, Japan), the following variables were assessed: total body fat (%), fat-free mass (kg), skeletal muscle mass (kg), total body water (kg and %), segmental fat and lean mass (arms, legs, trunk), visceral fat rating (arbitrary units), basal metabolic rate (kcal/day), and phase angle (°)—these parameters are well-established indicators of nutritional status and metabolic health.

The device has demonstrated high test–retest reliability with intraclass correlation coefficients (ICCs) ≥ 0.95 for total fat mass and total body water, compared to DXA standards. Measurement error is minimal (±0.1 kg for weight; ≤2% for body composition variables), suggesting residual variation is random rather than systematic.

### 2.11. Statistical Analysis

The database was created in Microsoft Excel 2010. Numerical variables are presented as mean ± standard deviation (SD). The normality of data distribution was assessed using the Shapiro–Wilk test. Based on the results, either parametric (*t*-tests) or non-parametric tests were applied, specifically the Wilcoxon test for paired data. We calculated the differences between the baseline values and those obtained after the treatment, and based on these differences, we conducted a one-way ANOVA to assess whether the changes were significantly influenced by sex or by age group (<40 years vs. >40 years). Statistical analyses were performed using GraphPad Prism (version 10). The set confidence threshold was 95% (*p* < 0.05).

## 3. Results

Of the 77 participants, 58 were female (28–55 years) and 19 were male (20–49 years).

The combined intervention between Eximia body remodeling (RD) and muscle stimulation (MS), resulted in significant changes in muscle mass (T0 = 37.34 vs. T1 = 38.13; *p* = 0.02), extracellular water (T0 = 15.46 vs. T1 = 15.28; *p* = 0.04), percentage fat (T0 = 26.11 vs. T1 = 25.04; *p* = 0.04), weight (T0 = 71.24 vs. T1 = 70.02; *p* = 0.024), and fat expressed in Kilograms (T0 = 19.21 vs. T1 = 18.19, *p* = 0.01) (Table 1).

In women, the combined intervention of Eximia body remodeling (RD) and muscle stimulation (MS) had the potential to influence extracellular water (T0 = 14.87 vs. T1 = 14.6; *p* = 0.007), body mass index (T0 = 26.16 vs. T1 = 25.72; *p* = 0.004) and weight (T0 = 72.27 vs. T1 = 69.31; *p* = 0.006), (Table 2).

For men, the combined intervention of Eximia body contouring and EMS influenced muscle mass % (T0 = 42.17 vs. T1 = 42.72; *p* = 0.041) (Table 3).

Gender-based comparisons revealed that female participants exhibited greater improvements in fat mass (kg), body weight, BMI, body fat percentage, and extracellular water (*p* < 0.05), whereas male participants demonstrated significant change only in muscle mass percentage.

### Age Analyses

Age 40 emerged as a relevant threshold for subgroup analysis based on observed outcome variability; accordingly, body mass index (BMI) differed significantly before versus after treatment (*p* = 0.027). The following are shown: bio-impedance (T0 = 594.99 vs. T1 = 599.58; *p* = 0.02), body mass index (T0 = 26.03 vs. T1 = 25.53; *p* = 0.027), weight T0 = 70.64 vs. T1 = 69.09; *p* = 0.002), fat expressed in kg (T0 = 18.32 vs. T1 = 17.02; *p* = 0.0025) (Table 4).

The intervention implemented in this experiment was specifically designed to elucidate the effect of age on the measured outcomes.

Age subgroup analysis revealed significant differences in response to the intervention. Participants over 40 years of age showed statistically significant improvements in extracellular water (*p* = 0.07), impedance (*p* = 0.02), weight (*p* = 0.004), and body mass index (BMI) (*p* = 0.027).

The difference in BMI between the two time points was not significantly influenced by sex (*p* = 0.080), although the result suggests a trend toward statistical significance. A similar pattern was observed for fat mass, where the differences were not statistically significant (*p* = 0.819) (Table 5).

## 4. Discussion

The present study demonstrated favorable shifts in body composition following the combined Eximia + EMS intervention, notably through a significant reduction in fat mass. This reduction led to an increase in the percentage of muscle mass, without a significant change in absolute muscle mass (kg), underscoring the distinction between relative and absolute metrics of body composition. Since muscle mass percentage is calculated as a proportion of total body mass, a decrease in the denominator (mainly due to fat loss) naturally increases this percentage even if muscle mass in kilograms remains stable.

This compositional improvement aligns with the intended goals of non-invasive body contouring strategies aimed at reducing fat and improving anthropometric parameters and reflects prior evidence suggesting EMS can be effective in altering body composition [9,10,11]. The body-shaping effects observed in our participants are consistent with those reported in other studies involving similar technology, where improvements in physical and physiological markers have been recorded after EMS-based interventions [11].

Subgroup analysis indicated a differentiated response to the intervention between genders. Women exhibited broader changes in overall body composition, while men showed significant increases in percent muscle mass only. These differences may reflect previously documented variations in neuromuscular response to EMS, as women have shown increased surface excitability and sensory sensitivity to stimulation [12]. Such gender-specific responses suggest that EMS and similar interventions might yield varying outcomes depending on sex and should be interpreted accordingly [13,14,15,16].

Participants over the age of 40 experienced significant improvements in parameters such as extracellular water, impedance, weight, and BMI, whereas those under 40 did not show significant changes. These findings suggest a potentially greater responsiveness to the intervention among older individuals, possibly related to age-related changes in tissue composition or metabolic profile [17]. However, since no hormonal or biochemical markers were collected in this study, interpretations involving endocrine factors must remain speculative and beyond the scope of this data set.

Further controlled studies are warranted to assess whether age influences the efficacy of such interventions, especially under standardized nutritional and physical activity conditions. It is important to note that participants also received structured nutritional education and reported increased physical activity, which may have contributed to the observed improvements as lifestyle factors are known to enhance body composition outcomes [18].

The study employed rigorous standardization protocols—10 h fasting and hydration prior to each session—to reduce variability and improve the precision of bioelectrical impedance analysis (BIA) measurements. Proper hydration improves current conductivity, while fasting reduces acute metabolic fluctuations that could affect impedance values [19,20,21].

A decrease in impedance is commonly interpreted as a marker of improved body composition, typically reflecting reductions in adipose tissue and/or enhanced tissue hydration—both of which are influenced by intracellular and extracellular water content, as well as the electrical conductivity of bodily tissues [22,23,24]. BIA also allows tracking of extracellular water (ECW), intracellular water (ICW), and their ratio, all of which contribute to understanding changes in tissue structure and inflammation status [25,26,27]. A decrease in ECW% may suggest reduced water retention or inflammation, but again, in the absence of inflammatory biomarkers, such interpretations must be limited.

Mild side effects—such as localized redness due to Eximia—were noted but did not affect participation and are known transient effects of mechanical or thermal skin stimulation [28].

The decision to employ a pre-post single-cohort design was based on ethical and practical considerations. Withholding a potentially beneficial intervention from a control group raised concerns, especially since this was an exploratory study conducted in a clinical setting. Additionally, logistical limitations made the inclusion of an untreated cohort impractical during the study window.

To enhance internal validity, participants served as their own controls, with standardized behavior tracking (food records, IPAQ-SF) and protocol adherence. By maintaining consistent fasting, hydration, and post-session practices, we minimized inter-subject variability and enhanced the reliability of within-subject comparisons.

The Tanita MC-780MA-N device used in this study applies multifrequency currents (5–1000 kHz) to estimate segmental body composition. Calculations are based on resistance, reactance, and proprietary regression algorithms that incorporate participant-specific variables such as age, sex, and height. The phase angle—recorded at 50 kHz—was included as an index of cell membrane integrity and nutritional status.

Device validation studies report high reliability for measurements of fat mass, body water, and phase angle, with intraclass correlation coefficients (ICCs) above 0.90 when compared to dual-energy X-ray absorptiometry (DXA) [25]. Standardization of measurement conditions (hydration, fasting, bladder emptying, ambient temperature, and measurement timing) contributed to consistency and reliability across sessions.

Due to the nature of multifrequency BIA, changes in ECW% or fat% may occur even in the absence of significant shifts in total impedance, especially when ICW:ECW ratios are affected. Likewise, segmental fluctuations in reactance (Xc) or minor changes in the underlying regression models may influence fat% estimates without materially altering global impedance (Z) within its margin of error [15,29].

## 5. Practical Applications

The favorable changes in body composition observed following the combined Eximia and EMS protocol emphasize the potential of integrating non-invasive body contouring technologies within comprehensive, multidisciplinary lifestyle interventions. From a nutritional perspective, the incorporation of structured dietary education and standardized peri-procedural nutritional guidance likely contributed to the reduction in adiposity and the preservation of lean mass. These findings reinforce the critical role of individualized nutritional strategies as synergistic components that enhance the physiological impact of technological interventions.

In both clinical and wellness contexts, the deployment of multimodal protocols combining localized electrostimulation, non-invasive lipolytic procedures, and personalized nutritional counseling may offer an effective approach to body recomposition. Such integrative interventions support not only aesthetic outcomes but also metabolic health, particularly when adapted to the specific needs and behavioral patterns of diverse populations. Furthermore, evidence suggests that specific interventions, through educational updates and increased support, can improve health outcomes and the trust placed in healthcare professionals’ recommendations continues to be instrumental [30,31]. In this context, a formalization of the intervention process is particularly valuable, as it enables a clearer understanding of both its inherent limitations and potential benefits, as highlighted in recent studies [32,33].

### Limitations

To more robustly support the observed associations, future studies should include a larger participant sample. The present investigation relied on a convenience sample which, although it revealed biologically plausible effects of potential scientific interest, lacked the statistical power required to reach conventional significance thresholds; therefore, the findings should be interpreted as preliminary. In addition, body-composition assessment was performed with a Tanita bioimpedance scale; despite its classification as a medical device, bioimpedance analysis remains an indirect technique whose accuracy can be affected by hydration status, recent food or fluid intake, skin temperature and electrode placement, thereby introducing measurement error and further limiting the precision of the reported outcomes.

## 6. Conclusions

The combined application of Eximia and EMS interventions resulted in clinically meaningful improvements in body composition, including reductions in fat mass and extracellular water, alongside significant gains in skeletal muscle mass—effects particularly pronounced in participants aged over 40. For sedentary adults at increased risk of lifestyle-related metabolic disorders, these non-invasive modalities, when integrated with tailored nutritional and physical activity guidance, offer a feasible and health-focused therapeutic strategy. This standardized and user-compliant protocol has the potential to enhance metabolic function, support nutritional status, and improve overall quality of life, with aesthetic outcomes positioned as a secondary, yet motivating, benefit.

## Figures and Tables

**Table 1:** Comparison Before and After the Combined Eximia & EMS Intervention.

Variable	Before		After		*p* Value
	Mean	SD	Mean	SD	
Muscle mass %	37.34	5.09	38.13	5.94	0.02 *
Muscle mass in kg	25.88	5.04	26.71	4.487	0.74 **
Phase Angle	6.22	0.61	6.40	0.64	0.16 *
Intracellular water	19.78	3.39	19.96	3.29	0.72 **
Extracellular water	15.46	2.12	15.28	2.09	0.04 *
Metabolic age	36.00	12.79	35.97	12.78	0.95 *
Bone density	2.61	0.34	2.61	0.33	0.99 *
Visceral fat	4.97	2.88	4.74	2.99	0.08 *
Total body water	35.82	6.01	35.14	5.15	0.25 *
BMR kj	6457.65	876.69	6418.15	841.49	0.45 **
Bioimpedance	614.90	64.03	618.45	73.19	0.77 *
Fat-free mass	52.02	7.21	51.67	7.02	0.39 **
Fat %	26.11	8.13	25.04	9.02	0.04 *
BMI body mass index	26.03	4.26	25.68	4.16	0.054 *
Waist–hip ratio	0.87	0.07	0.87	0.07	0.38 *
Weight	71.24	12.37	70.02	11.75	0.024 *
Fat kg	19.21	8.23	18.19	8.42	0.01 *

* *t* Test, ** Wilcoxon test.

**Table 2:** The combined Eximia and EMS intervention for women.

Variable	Before		After		*p* Value
	Mean	SD	Mean	SD	
Muscle mass %	36.34	4.673	37.19	5.82	0.27 *
Muscle mass in kg	47.27	5.29	46.83	4.27	0.29 *
Phase angle	6.13	0.56	6.35	0.68	0.13 *
Intracellular water	18.41	1.37	18.59	1.18	0.47 *
Extracellular water	14.87	1.78	14.6	1.55	0.007 *
Metabolic age	35.41	12.44	35.52	12.12	0.84 *
Bone density	2.51	0.26	2.49	0.21	0.45 *
Visceral fat	4.48	2.4	4.24	2.49	0.16 **
Total body water	33.97	4.63	33.06	2.29	0.12 **
BMR kj	6193	684.1	6122	552.2	0.12 *
Bioimpedance	621.3	67.14	629.5	73.17	0.38 **
Fat-free mass	49.78	5.55	49.13	4.46	0.13 *
Fat %	27.08	8.26	25.98	9.16	0.06 **
BMI body mass index	26.16	4.48	25.72	4.39	0.004 *
Waist–hip ratio	0.85	0.06	0.84	0.06	0.098 *
Weight	72.24	12.37	69.31	12.21	0.003 **
Fat kg	19.53	8.63	18.41	8.75	0.014 *

* *t* Test, ** Wilcoxon test.

**Table 3:** Eximia and EMS combined intervention for men.

Variable	Before		After		*p* Value
	Mean	SD	Mean	SD	
Muscle mass %	42.17	4.56	42.72	4.43	0.041 *
Muscle mass in kg	59.75	3.12	60.75	2.25	0.29 *
Phase angle	6.70	0.63	6.65	0.29	0.75 *
Intracellular water	26.43	1.99	26.62	1.35	0.64 *
Extracellular water	18.37	0.88	18.6	0.72	0.20 *
Metabolic age	38.83	15.33	38.17	16.77	0.61 *
Bone density	3.13	0.15	3.2	0.10	0.23 *
Visceral fat	7.33	4.03	7.16	4.30	0.61 *
Total body water	44.8	2.82	45.22	2.05	0.45 *
BMR kj	7734	494.9	7850	369.8	0.46 **
Bioimpedance	584.2	35.68	565.1	48.29	0.14 *
Fat-free mass	62.88	3.27	63.95	2.35	0.29 *
Fat %	21.47	6.02	20.55	7.37	0.32 *
BMI body mass index	25.38	3.22	25.55	3.12	0.61 *
Waist–hip ratio	0.96	0.04	0.97	0.04	0.48 *
Weight	80.6	8.93	81.08	8.46	0.64 *
Fat kg	17.72	6.31	17.13	7.41	0.48 *

* *t* Test, ** Wilcoxon test.

**Table 4:** Age Group-Based Comparison of Outcomes Before and After the Combined Eximia and EMS Intervention.

Parameters	>40 Years (*n* = 35)					<40 Years (*n* = 42)				
	Before		After		*p* Value	Before		After		*p* Value
	Mean	SD	Mean	SD		Mean	SD	Mean	SD	
Muscle mass %	36.92	5.23	37.55	5.79	0.58 *	37.68	5.09	38.62	6.17	0.18 *
Muscle mass in kg	26.44	4.98	25.85	3.81	0.71 *	38.62	5.20	27.25	4.96	0.91 **
Phase Angle	6.37	0.67	6.51	0.62	0.48 *	6.10	0.53	6.30	0.65	0.30 *
Intracellular water	19.53	3.01	19.79	2.84	0.67 **	19.99	3.75	20.10	3.70	0.97 **
Extracellular water	15.44	2.07	15.18	1.99	0.07 *	15.48	2.22	15.37	2.23	0.32 *
Metabolic age	37.56	10.75	37.62	10.78	0.91 *	34.68	14.45	34.57	14.4	0.89 *
Bone density	2.63	0.30	2.61	0.29	0.68 *	2.61	0.37	2.61	0.37	0.85 *
Visceral fat	5.43	2.92	5.12	3.05	0.13 *	4.57	2.87	4.42	2.98	0.38 *
Total body water	36.22	6.36	34.73	4.61	0.18 **	35.48	5.84	35.48	5.67	0.89 *
BMR kj	6419.46	804.19	6362.42	738.42	0.37 *	6489.82	954.18	6465.08	937.05	0.45 **
Bioimpedance	594.99	49.31	599.58	52.75	0.02 *	631.67	71.20	634.34	84.95	0.98 **
Fat-free mass	52.32	6.51	51.65	6.15	0.26 *	51.77	7.92	51.68	7.84	0.92 **
Fat %	70.64	12.08	69.09	804.19	0.37*	25.05	8.66	26.25	9.35	0.25 *
BMI body mass index	26.03	4.39	25.53	4.03	0.027 *	25.96	4.40	25.61	3.93	0.46 *
Waist–hip ratio	0.88	0.07	0.88	0.07	0.85 *	0.86	0.08	0.86	0.07	0.20 *
Weight	70.64	12.55	69.09	12.08	0.004 *	71.74	12.53	70.81	11.74	0.20 *
Fat kg	18.32	8.05	17.08	8.57	0.025 *	19.96	8.52	19.13	8.41	0.10 *

* *t* Test, ** Wilcoxon test.

**Table 5:** Gender- and age-specific responses to non-invasive body-contouring interventions.

Variable	Group	*p* Value
BMI_Diff	Gender	0.080
BMI_Diff	Age	0.750
Muscle_Mass_Diff	Gender	0.185
Muscle_Mass_Diff	Age	0.643
Fat_Mass_Diff	Gender	0.212
Fat_Mass_Diff	Age	0.819
Fat_Free_Mass_Diff	Gender	0.089
Fat_Free_Mass_Diff	Age	0.680
Visceral_Fat_Index_Diff	Gender	0.240
Visceral_Fat_Index_Diff	Age	0.974

## Data Availability

The data presented in this study are available on request from the corresponding author. The data are not publicly available due to privacy and ethical restrictions, as they contain sensitive health-related information from human participants that cannot be disclosed in compliance with applicable confidentiality and data protection regulations.
